# Hepatoprotective activity of *Moringa oleifera* against cadmium toxicity in rats

**DOI:** 10.14202/vetworld.2015.537-540

**Published:** 2015-04-24

**Authors:** Reetu Toppo, Birendra Kumar Roy, Ravuri Halley Gora, Sushma Lalita Baxla, Prabhat Kumar

**Affiliations:** Department of Veterinary Pharmacology & Toxicology, College of Veterinary Science & A. H., Birsa Agricultural University, Ranchi - 834 006, Jharkhand, India

**Keywords:** cadmium, *Moringa oleifera*, rats, serum bio-markers, toxicity

## Abstract

**Aim:**

The present investigation has been conducted to evaluate the hepatoprotective activity of *Moringa oleifera* against cadmium-induced toxicity in rats.

**Materials and Methods:**

For this study, 18 Wistar albino rats were taken. Control group, Group I rats were given cadmium chloride @ 200 ppm per kg and Group II rats were treated with *M. oleifera* extract @ 500 mg/kg along with cadmium chloride @ 200 ppm per kg (daily oral for 28 days). On 29^th^ day, animals were slaughtered and various parameters were determined. Serum biomarkers, oxidative stress parameters, histomorphological examination were carried out with estimation of cadmium concentration in liver tissues.

**Results:**

Oral administration of cadmium chloride @ 200 ppm/kg for 28 days resulted in a significant increase in aspartate aminotransferase (AST), alanine transaminase (ALT), alkaline phosphatase (ALP), significant (p≤0.01) increase of lipid peroxidation (LPO) and decrease in superoxide dismutase (SOD), and increase in cadmium accumulation in liver. Treatment with *M. oleifera* @ 500 mg/kg significantly (p<0.01) decreased the elevated ALP, AST, ALT, LPO levels and increase in SOD levels, and as compared to cadmium chloride treated group. However, there was no significant difference in cadmium concentration in liver when compared with cadmium chloride treated group.

**Conclusion:**

The study conclude that supplementation of *M. oleifera* (500 mg/kg), daily oral for 28 days has shown protection against cadmium-induced hepatotoxicity.

## Introduction

The exposure to the toxic metals has become an increasingly recognized source of illness worldwide [1,2]. Cadmium is a well-known heavy metal present in the environment and causes serious environmental and occupational hazard to human [3,4]. Cadmium induces lipid peroxidation by stimulating the production of superoxide anions and inhibits antioxidants such as glutathione peroxidase and superoxide dismutase and cause accumulation of free radicals that damage the cells and produce chronic disease [5]. Medicinal plants are the backbone of traditional medicine, which means more than 3.3 billion people in the less developed countries, utilizes medicinal plants on a regular basis [6]. *Moringa oleifera* is referred as “Miracle tree” in tropics and subtropics with a wide range of the beneficial effect, which was predicted in Indian system of medicine (Ayurveda and Unani) [7]. The plant is reported to possess antitumor, antipyretic, anticonvulsant, anti-inflammatory [8], antiulcer, antispasmodic, antidiabetic, diuretic, antihypertensive, antioxidant antifungal, antibacterial [9,10], antiretroviral, antisepticemic, antidiarrheal, and can be used to treat hepatorenal, cardiovascular, gastrointestinal, and hematological disorders [11], anxiety, asthma, bronchitis, cough, conjunctivitis, arthralgia, psoriasis, and diabetes [12].

There were less evidences regarding efficacy of *M. oleifera* against cadmium-induced toxicity, keeping in mind we conducted this study to evaluate the hepatoprotective activity of *M. oleifera*.

## Materials and Methods

### Ethical approval

The protocol of the experiment was approved by the Institutional Animal Ethics Committee with approval number 134/IAEC/RVC and protocols were followed according to the guidelines given by Committee for the Purpose of Control and Supervision of Experiments on Animals.

### Experimental animals

A total of 18 Wistar albino rats were taken and randomly divided into three groups (n=6). They are housed in propylene cages under standard laboratory conditions with standard food and water ad-libitum. For this study, control in which healthy rats were given standard feed and deionized water, Group I rats were given cadmium chloride @ 200 ppm per kg daily oral for 28 days and Group II rats. *M. oleifera* leaf extract @ 200 mg/kg along with cadmium chloride @ 200 ppm per kg orally daily for 28 days.

### Chemicals

Cadmium chloride was dissolved in distilled water and given orally to individual animal @ 200 ppm [13].

### Plant extract

The leaves of *M. oleifera* were collected, washed with distilled water, shade-dried, pulverized and freshly prepared powder (25 g) were immersed in hydro-alcoholic solution (40% distilled water + 60% ethanol) in a flask stoppered and was kept at room temperature for 48 h at 150 rpm in orbital shaker. The contents were filtered through muslin cloth and filtered through whatman No. 1 filter paper and extract dried in a Petri dish at room temperature and used along with gum acacia @ 500 mg/kg [14] was given to individual animal with oral gavage needle.

### Sample collection

Blood samples were collected into ethylenediaminetetraacetic acid vials as well as in plain vials on day 29 from the heart puncture. Serum was separated by following standard procedure and was kept in the refrigerator at 4°C till analysis. Liver was collected and kept in −20°C for the estimation of oxidative stress parameters and cadmium concentration.

### Biochemical estimation

Aspartate aminotransferase (AST), alanine transaminase (ALT) and alkaline phosphatase (ALP) in serum were analyzed using Erba Semi-auto analyzer by Erba-biochemkits.

### Oxidative stress parameters

The pieces of liver thus collected after the sacrifice of the experimental animals were washed in ice cold saline and 200 mg of liver tissue sample was weighed and taken in 2 ml of ice-cold saline. The homogenate was prepared in Remi-Homogeniser and was centrifuged at 3000 rpm for 10 min. The supernatant was used for the estimation of following oxidative stress indices. Superoxide dismutase was estimated [15]. The extent of lipid peroxidation was evaluated in terms of malondialdehyde production, determined by the thiobarbituric acid method [16].

### Cadmium concentration

Cadmium concentration was quantitatively analyzed on day 29 liver tissue with the help of AAS [17]. The tissue sample was digested using acid and filtrate was prepared by using Millipore water. Then final volume was made up to 10 ml with Millipore water for reading on An Elico double beam atomic absorption spectrophotometer SL 176 model used for total cadmium estimation. Working standards were prepared by dilution of stock (1000 µg/ml) and intermediate (30 µg/ml) standards. The working standards were as follows: 0, 0.2, 0.5, 0.8, 1, 10, 20, 30 µg/ml and prepared it by the same procedure as a test.

### Statistical analysis

Quantitative data were analyzed, using the ANOVA. A value p≤0.05 and p≤0.01 were considered significant at 5% and 1%, level respectively.

## Results

### Serum bio-markers

It is evident from Table-1 that serum biomarker levels of ALT, AST, and ALP were significantly (p<0.01) higher in both Groups II and III compared to Group I, though the activities of enzymes were significantly (p<0.01) lower in Group III compared to Group II.

**Table-1:** Effect of *Moringa oleifera* against cadmium treated groups on various parameters (mean±SE) (n=6).

Parameters	Control	Group I	Group II
ALT (I.U/L)	37.75±0.88	69.72±0.85**	51.57±0.89^A^**
AST (I.U/L)	187.03±1.43	224.90±1.21**	191.47±0.86^A^**
ALP (I.U/L)	113.53±1.04	152.26±1.53**	134.64±2.03^A^**
LPO (Nm MDA g^−1^)	1.45±0.21	3.44±0.21**	2.74±0.26^A^*
SOD (U/mg of protein)	4.65±0.32	2.93±0.18**	3.69±0.22^A^**
Liver (ppm)	0.09±0.02	19.28±0.41**	17.30±1.49^NS^

** p<0.01;

*p<0.05; statistically significant when compared to control group,

A* p<0.05;

A** p<0.01;

statistically significant when compared to Group I, NS=Statistically non-significant when compared with Group I, LPO=Lipid peroxidation, AST=Aspartate aminotransferase, ALT=Alanine transaminase, ALP=Alkaline phosphatase, SOD=Superoxide dismutase, SE=Standard error, *M. oleifera=Moringa oleifera*

### Oxidative stress indices

It is transpired from Table-1 that lipid peroxidation in liver tissue was significantly (p<0.01) higher in only cadmium treated group (Group II) whereas *M. oleifera* treated animals (Group III) showed significant (p<0.05) decrease in lipid peroxidation compared to the Group II. Superoxide dismutase (SOD) level in liver was significantly (p<0.01) lower in both Group I and Group II compared to control group, though the level was again significantly (p<0.01) higher in Group II compared to Group I (Table-1).

### Cadmium concentration

At the end of the experiment on 29^th^ day, there was significant (p<0.01) increase in cadmium concentration in liver tissue of Group I and Group II as compared to the control group (Table-1). There was no significant difference in cadmium concentration in Group II as compared with Group I.

## Discussion

Cadmium is a non- essential trace element which is toxic to many plants and animals. Large numbers of enzymatic activities are influenced by cadmium and the mechanism of this effect has been hypothesized to be due to their displacement of a beneficial metal from the active site in the enzyme itself [18]. Exposure to cadmium for a short period of time affects the liver [19]. In the present study, there was a significant increase in ALT, ALP, and AST levels were observed (Table 1) indicates liver damage. Elevated serum levels ALT, ALP, and AST may be due to hepatocellular necrosis, which caused increase in the permeability of the cell membrane resulting in the release of transaminases in the blood stream. The increase in alkaline phosphatase activities represent general hepatic toxicity [20]. Induction of alkaline phosphates synthesis is the usual response of the liver to any form of biliary obstruction [21]. Similar finding was observed by [22-28]. There was significant decrease in ALT, ALP, and AST levels when co-treated with cadmium and *M. oleifera* (Table-1). It might be due to hepatoprotective activity. The reversal of elevated serum intracellular enzyme levels by MO extract may be attributed to the stabilizing ability of the cell membrane preventing enzyme leakages as earlier postulated [29] and might be due to hepatoprotective property of *M. Oleifera* leaf extract. Previous study reported hepatoprotective effect was due to presence of Quercetin and kaempferol [30]. In the present study, there was increase in lipid peroxidation and decrease in SOD was observed in rats of cadmium chloride treated group (Table-1). It correlates with the studies of [31-34]. This might be due to the peroxidation of membrane lipids and injury to the cellular components.

Reduction in lipid peroxidation and increase in SOD level was observed when cadmium was co-treated with *M. oleifera* (Table-1). It might be due to the presence of flavonoids such as quercetin and kaempferol, vitamin A, ascorbic acid. Ascorbic acid is considered as a potent antioxidant. Similar findings were observed by [35-38]. Significant increase in cadmium level in liver was observed in cadmium treated rats (Group I) and (Group II) as compared to control the group. It is generally known that cadmium is mainly accumulated in kidneys and liver [39] because these organs contain most of metallothionein binding toxic metals [40]. Study proves that exposure to cadmium for 28 days causes toxicity in the body. It was confirmed by the results shown in this study.

## Conclusion

*M. oleifera* possess antioxidant and free radical scavenging property, which could be helpful in reducing the oxidative stress caused by cadmium, by reducing the ROS production, maintaining the antioxidant potential, and significantly reducing elevated serum biomarker levels in the body. Our study shows that supplementation of *M. oleifera* extract (@ 500 mg/kg) showed hepatoprotective effect against cadmium toxicity.

## Authors’ Contributions

BKR supervised the overall research work. RT, RHG, PK, SLB participated in research work, analysed the samples, statistically analysed the results, modified the article and made available relevant literatures. All authors read and approved the final manuscript.
